# Unveiling the Silent Threat of Upper Cervical Disc Herniation: A Case Report

**DOI:** 10.7759/cureus.83048

**Published:** 2025-04-26

**Authors:** Kein Yoshimura, Hirohito Hirata, Masatsugu Tsukamoto, Yu Toda, Tadatsugu Morimoto

**Affiliations:** 1 Department of Orthopedic Surgery, Saga University, Saga, JPN

**Keywords:** cervical cord, differential diagnosis, intervertebral disc displacement, joint dislocations, laminoplasty, neurologic manifestations

## Abstract

Traumatic herniations of the upper cervical spine are rare, with a higher likelihood of occurrence in older individuals. Their associated neurological symptoms can vary widely, often leading to delays in clinical diagnosis. We report the case of an 86-year-old man who developed neurological symptoms after a fall. Initially, intracranial pathology was suspected, and a head magnetic resonance imaging (MRI) revealed small chronic subdural hematomas, which were managed conservatively. However, as his paralysis progressed over the following days, a repeat brain CT showed no significant changes. Suspecting cervical spine involvement, further imaging identified a C2/3 disc herniation. The patient underwent emergency cervical laminoplasty, but postoperative subluxation required additional surgery, including C1-3 posterior fusion and C2/3 anterior fusion. Post-surgery, the patient exhibited improvement in paralysis affecting both the upper and lower extremities and a reduction in sensory deficits. Early diagnosis and treatment are crucial to improve neuropathic outcomes. A thorough understanding of the symptoms and characteristics of neurological damage to the upper cervical spine can significantly contribute to favorable results. Clinicians should be well-acquainted with this pathological condition.

## Introduction

Cervical intervertebral disc herniations commonly manifest between the ages of 30 and 50 years, occasionally occurring suddenly without apparent cause. Although they frequently occur at the lower levels (C5/6 and C6/7), herniations at the C2/3 level are rare, less than 1% [1-3]. In addition, cervical disc herniations at the upper levels are more common in older adults [3-5]. Symptoms of lower-to-middle cervical disc herniation typically have characteristic manifestations depending on the affected nerve roots or spinal cord. However, such upper-level herniations are especially difficult to diagnose because their symptoms - like headache, limb weakness, or numbness - can mimic conditions originating in the brain (e.g., stroke or hematoma). This diagnostic challenge can lead to delayed or inappropriate treatment. Furthermore, such delays may contribute to prolonged symptoms and increased patient burden. Herein, we report a rare case of C2/3 disc herniation in an elderly patient that was initially misdiagnosed as a brain disorder. This case underlines the need for careful clinical assessment and timely imaging, and we discuss it in light of existing literature.

## Case presentation

Case history

An 86-year-old male suffered a fall impacting the posterior aspect of his head. Subsequently, the patient reported discomfort characterized by head and neck pain, reduced stability while walking, and compromised fine motor control in his right hand, significantly impeding mobility. His medical history included atrial fibrillation and cerebral infarction necessitating warfarin administration.

**Figure 1:**
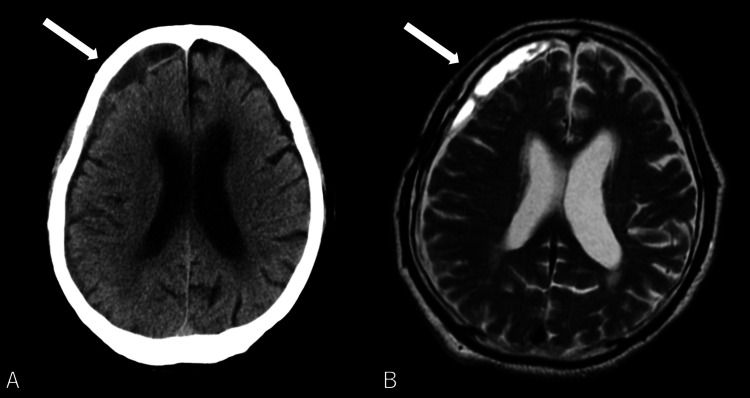
Brain computed tomography scan (A) and magnetic resonance imaging (B) showing a chronic subdural hematoma in the right frontal region (arrows).

Upon immediate response, the patient was promptly transported by ambulance to the neurosurgery department of a local hospital, where a thorough examination was conducted. The initial assessment suggested intracranial pathology due to trauma. Subsequent brain computed tomography (CT) and magnetic resonance imaging (MRI) confirmed the presence of a chronic subdural hematoma (Figures 1A-1B), identified as the likely cause of the observed symptoms. Therefore, conservative management was initiated. During hospitalization, the patient experienced a gradual onset of extremity weakness.

In the days following the injury, due to the progressive nature of paralysis, a repeat brain CT was performed, revealing no discernible changes in the imaging findings. Given this clinical trajectory and the potential presence of cervical spine pathology, an imaging study of the cervical spine was conducted, revealing disc herniation at the C2/3 level. The patient was subsequently referred to our orthopedic department for surgery.

Diagnosis and treatment

Upon initial consultation, the patient claimed a slight neck pain and the manual muscle test (MMT) indicated a strength level of 4/5 in both the upper and lower limbs. Over time, the paralysis intensified, and muscle strength decreased to 1/5, but respiratory function remained intact. Additionally, the patient reported hypoalgesia and hypoesthesia in both the upper and lower limbs below the knees, while the deep sensation remained intact. Notably, the triceps, biceps, and radial reflexes were absent, except for the right patellar tendon reflex, which remained intact.

**Figure 2:**
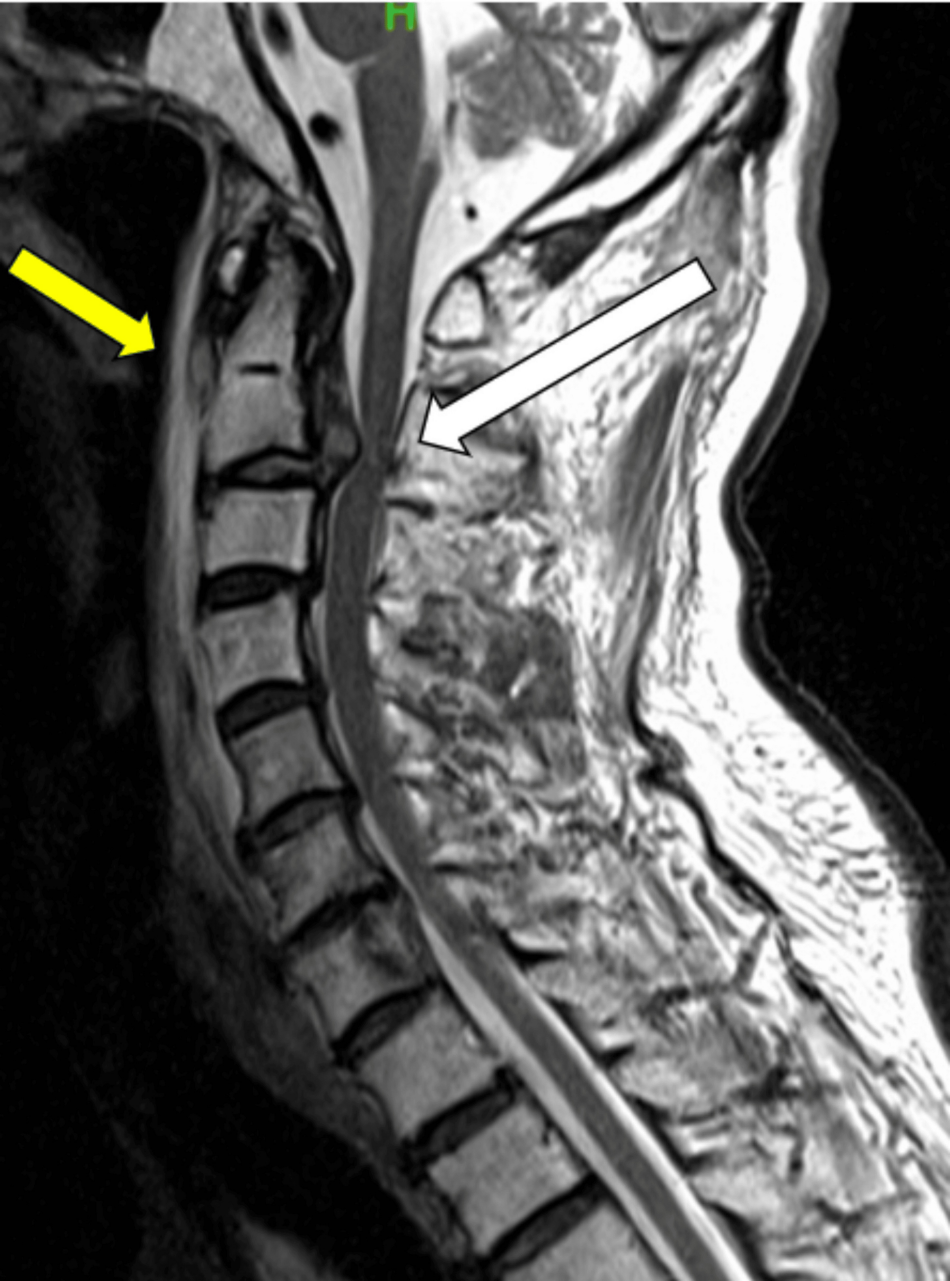
Sagittal T2-weighted MRI revealing a C2/3 disc herniation with cord compression (white arrow) and the pre-vertebral hematoma (yellow arrow) on the anterior aspect of the C2-5 vertebral body upon patient admission.

**Figure 3:**
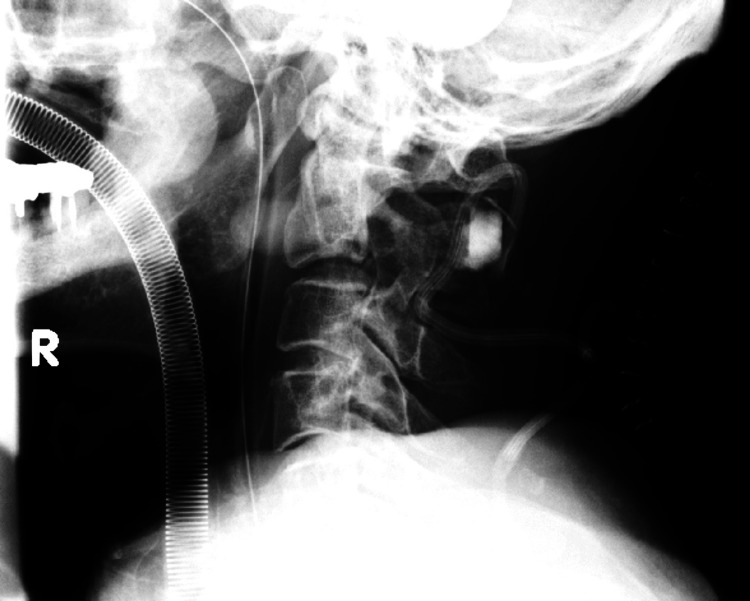
Plain lateral cervical X-ray after cervical laminoplasty showed a kyphotic angle of 10 degrees at the C2/3 level.

MRI performed in our department showed no abnormalities in the cranial region but confirmed the presence of a C2/3-disc herniation, along with a hematoma on the anterior aspect of the vertebral body spanning from C2 to C5, accompanied by a mild kyphotic deformity (Figure 2). Subsequently, emergency cervical laminoplasty of C2 and partial resection of the C3 lamina were performed. Immediately post-surgery, the patient demonstrated significant improvement, with the MMT levels escalating to grade 2 in the upper extremities and grade 3 in the lower extremities (Figure 3).

Outcome and follow-up

**Figure 4:**
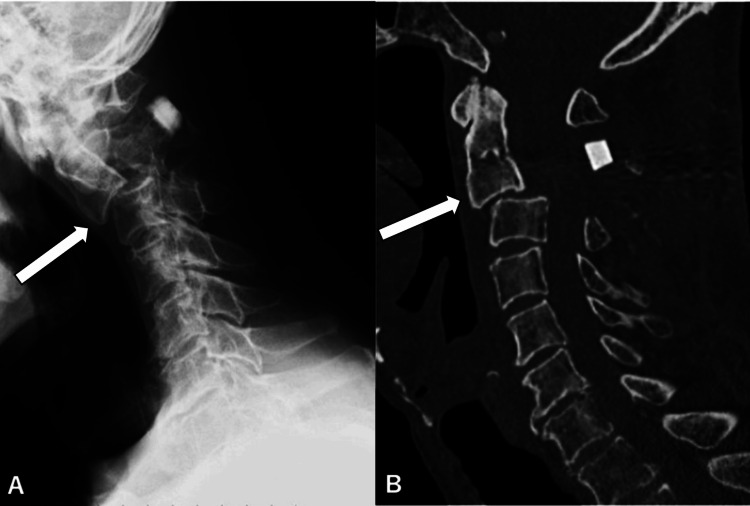
Plain X-ray (A) and CT (B) of the cervical spine on the same patient taken one week postoperatively, demonstrating subluxation at the C2/3 level, the kyphotic angle of 33 degrees (arrows).

**Figure 5:**
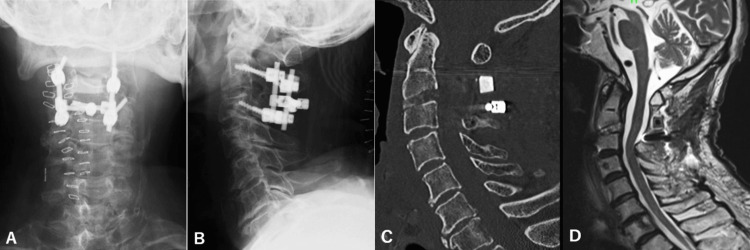
Plain anterior-posterior (A) and lateral (B) X-rays and CT (C) on the same patient after completion of anterior and posterior fusion, showing realignment of his cervical spine, the kyphotic angle of 3 degrees. MRI (D) revealing complete decompression of the spinal cord.

One week after the initial surgical intervention, the patient reported exacerbated neck pain, prompting further imaging that revealed subluxation at the C2/3 level (Figures 4A-4B). Consequently, the patient underwent corrective surgery targeting the subluxation involving simultaneous anterior and posterior cervical fusions at the C2/3 level (Figures 5A-5D). Following this procedure, the patient exhibited further enhancement of limb strength and a reduction in sensory disturbances. Over the subsequent days, patient progression continued.

At the last follow-up, conducted two weeks postoperatively, the patient demonstrated substantial recovery, achieving a strength rating of 4/5 for all four limbs. Importantly, full sensory function was restored, with only residual fine motor skill impairments in the right hand.

Summary of this case

An 86-year-old man presented with neck pain, gait instability, and decreased fine motor control in his right hand following a fall. Initially diagnosed with a chronic subdural hematoma and treated conservatively, he later developed progressive limb weakness. Imaging revealed a C2/3 disc herniation with anterior spinal hematoma. Despite initial improvement after decompressive surgery, the patient experienced worsening neck pain one week later, and imaging showed C2/3 subluxation. A corrective anterior-posterior fusion surgery was performed, leading to further neurological recovery. Two weeks postoperatively, the patient showed significant improvement in muscle strength and complete sensory recovery, with only mild residual fine motor impairment in the right hand (Table 1).

**Table 1:** Neurological timeline of the patient. ASIA: American Spinal Injury Association; MMT: manual muscle testing; UE: upper extremity; LE: lower extremity

Time point	ASIA classification	Key neurological findings	Diagnosis	Intervention	Outcome
Initial presentation (post-fall)	ASIA D	Neck and head pain, gait instability, decreased fine motor control in right hand, and MMT 4/5 in all limbs	Chronic subdural hematoma	Conservative management	Progressive limb weakness
During hospitalization	ASIA C	Worsening paralysis (MMT 1/5), hypoalgesia and hypoesthesia below the knees, preserved deep sensation, absent triceps, biceps, and radial reflexes	C2/3 disc herniation with anterior hematoma (C2-C5); mild kyphosis	Emergency C2 laminoplasty and partial C3 laminectomy	Improved motor strength (UE: MMT 2, LE: MMT 3)
1 week post-surgery	ASIA D	Increased neck pain and continued motor improvement	C2/3 subluxation	Anterior and posterior cervical fusion at C2/3	Further motor improvement and reduced sensory deficits
2 weeks post-operative (final follow-up)	ASIA D (improving)	Motor strength 4/5 in all limbs, full recovery of sensory function, and residual fine motor deficit in right hand	Postoperative status	Continued rehabilitation	Functional recovery nearly complete

## Discussion

Epidemiology

Cervical disc herniation predominantly occurs in the lower cervical segments, with disc herniation at the C2/3 level, the uppermost cervical disc space, being a rare occurrence and thus poorly described in the literature [2].

The lower cervical spine primarily facilitates flexion and extension movements of the head, subjecting these levels to the majority of loading forces during cervical spine movement. Conversely, the upper cervical spine, responsible for rotational movements of the head, experiences less strain [6]. This anatomical distinction contributes to the higher prevalence of cervical disc herniations around C5/6 and C6/7.

Notably, upper disc herniation is more frequently reported in older patients than in younger patients [3-5,7]. This observation is linked to spondylotic changes. Older patients often exhibit reduced mobility in the middle and/or lower cervical spine due to degenerative spondylosis alterations, potentially overloading the upper levels and causing upper cervical disc lesions [3].

Existing literature highlights that older individuals experiencing cervical spine trauma typically undergo stress on the upper cervical spine through analogous mechanisms [8,9]. In the specific context of this case, the traumatic event was postulated to have initiated stress on the C2/3 intervertebral disc, ultimately leading to disc herniation, despite the absence of concurrent fractures.

Symptoms

The differential diagnoses of neurological disorders can be broadly categorized into intracranial and spinal cord disorders. Common intracranial diseases include cerebral infarction, cerebral hemorrhage, epidural hematoma, and subdural hematoma, whereas spinal cord disorders encompass cervical disc herniation, trauma, and spinal neoplasms.

Nerve root and spinal cord injuries at the C2/3 level present with a variety of symptoms [10], including neck and radiating arm pain or numbness, sensory deficits, or motor dysfunction in the neck and upper extremities. Specific symptoms of nerve root disorders and typical muscle atrophy observed in lower cervical disc herniation were not present [11], making diagnosis solely based on symptoms challenging.

Moreover, C2/3 nerve disorders can lead to occipital headaches. The C3 pain dermatome encompasses a range of craniofacial areas, including the scalp above and behind the ear, the medial and lateral surfaces of the pinna, the lateral cheek over the angle of the jaw, the submental region, and the lateral and anterior aspects of the upper neck [12]. Compromised C3 nerve roots often result in patients complaining of pain and numbness due to occipital headaches.

In this case, myelopathy coexisting with intracranial disease was initially overlooked. However, as the symptoms persisted, cervical MRI was eventually performed, revealing disc herniation. This underscores the importance of considering high cervical disc herniation in the differential diagnosis when symptoms resembling intracranial disease are observed. Therefore, imaging of the cervical region should be considered, especially in the presence of cervical pain.

Treatment

Surgical approaches for C2/3 disc herniations vary, and the optimal treatment course remains a matter of debate [13]. The low frequency of cervical disc herniation at C2/3 and anatomical challenges are likely contributors to this debate.

In our case, due to the rapid progression of paralysis, emergency surgical intervention was required. Given our familiarity with the procedure, we initially performed laminoplasty. However, one week post-surgery, the patient's cervical pain worsened, and imaging revealed subluxation at C2/3. To address the subluxation, C2/3 anterior and posterior fusions were performed. An iliac bone autograft was used for anterior fixation following C2/3 discectomy. Subsequently, posterior fusion was achieved using C2 pedicle screws and C3 lateral mass screws, along with local bone grafting to the lamina.

The preoperative MRI already suggested a three-column injury [14], involving the posterior wall of the vertebral body, posterior longitudinal ligament, and posterior annulus fibrosus. Unfortunately, the need for fusion should have been identified and addressed during the initial surgery. Had dynamic X-rays or other additional tests been performed earlier, they might have provided a more comprehensive understanding of the injury and influenced the initial surgical plan. As a limitation of this case, the final follow-up was limited to an inpatient examination; therefore, long-term outcomes remain to be clarified.

Reminder to clinicians

Given the unique characteristics and challenges associated with high cervical disc herniation, clinicians must maintain a high index of suspicion, consider differential diagnoses for patients presenting with symptoms of intracranial disease, and conduct immediate evaluation. This involves comprehensive clinical assessment, adequate imaging studies, and multidisciplinary cooperation. MRI serves as a valuable diagnostic tool for both intracranial diseases and cervical disc herniation. A systematic evaluation, supported by appropriate imaging studies, also aids in minimizing the risk of complications. Concurrent performance of neck MRI with head MRI is recommended.

## Conclusions

In summary, this report highlights a rare case of C2/3 disc herniation initially misinterpreted as an intracranial pathology. Previous studies have described the diagnostic challenges of upper cervical disc herniation, often delayed due to nonspecific symptoms and a lack of early cervical imaging. In line with these reports, our case emphasizes the importance of including upper cervical disc herniation in the differential diagnosis when patients present with limb weakness, numbness, or headache, despite normal cranial imaging. This case contributes to the existing literature by reinforcing the need for heightened clinical suspicion and timely MRI evaluation in elderly patients with ambiguous neurological presentations.
